# Phonon-mediated high-*T*_*c*_ superconductivity in hole-doped diamond-like crystalline hydrocarbon

**DOI:** 10.1038/s41598-017-01541-6

**Published:** 2017-05-03

**Authors:** Chao-Sheng Lian, Jian-Tao Wang, Wenhui Duan, Changfeng Chen

**Affiliations:** 1 Beijing National Laboratory for Condensed Matter Physics, Institute of Physics, Chinese Academy of Sciences, Beijing, 100190 China; 2 School of Physics, University of Chinese Academy of Sciences, Beijing, 100049 China; 3 Department of Physics and State Key Laboratory of Low-Dimensional Quantum Physics, Tsinghua University, Beijing, 100084 China; 4 Department of Physics and High Pressure Science and Engineering Center, University of Nevada, Las Vegas, Nevada 89154 USA

## Abstract

We here predict by *ab initio* calculations phonon-mediated high-*T*_*c*_ superconductivity in hole-doped diamond-like cubic crystalline hydrocarbon *K*_4_-CH (space group *I*2_1_/3). This material possesses three key properties: (i) an all-*sp* ^3^ covalent carbon framework that produces high-frequency phonon modes, (ii) a steep-rising electronic density of states near the top of the valence band, and (iii) a Fermi level that lies in the *σ*-band, allowing for a strong coupling with the C-C bond-stretching modes. The simultaneous presence of these properties generates remarkably high superconducting transition temperatures above 80 K at an experimentally accessible hole doping level of only a few percent. These results identify a new extraordinary electron-phonon superconductor and pave the way for further exploration of this novel superconducting covalent metal.

## Introduction

Covalent metals are fascinating materials derived from doping selected semiconductors or insulators^1^. The strong directional bonding in these crystals produces high-frequency phonon modes that, when favorably coupled to charge carriers, are conducive to generating superconductivity at a high transition temperature *T*_*c*_. The prospects of finding light-element-based electron-phonon high-*T*_*c*_ superconductors have attracted reinvigorated research efforts in recent years^2–5^. Prominent among covalent metals are the carbon-based systems, whose structural versatility can be explored to tune the electronic, lattice, and electron-lattice coupling properties to optimize *T*_*c*_. Graphite and fullerene are prototypical *sp* ^2^-bonded carbon structures that become superconducting when doped with alkali or alkali-earth metals; examples include CaC_6_ with *T*_*c*_ = 11.5 K^6, 7^, and RbCs_2_C_60_ with *T*_*c*_ = 33 K^8, 9^ Superconductivity has also been observed in alkali-metal-doped aromatic hydrocarbons, e.g., K_3_ picene with *T*_*c*_ = 18 K^10^. Meanwhile, diamond is the archetype of *sp* ^3^-bonded carbon structures that turns into a superconductor with boron substitution. In contrast to the *sp* ^2^-bonded structures with *π* electrons as the main charge carriers^8, 10^, boron-doped diamond has holes injected into the *σ* states that respond sensitively to the strong bond-stretching phonon modes^11^. A similar electron-phonon coupling in the *σ*-band of MgB_2_ produced a *T*_*c*_ of 39 K^12, 13^. Doped diamond, however, has much lower *T*_*c*_ values in the range of 4 to 11 K at the boron concentration of 2.8 to 5%^14–16^.

According to the Bardeen-Cooper-Schrieffer theory of superconductivity^17^, achieving high *T*_*c*_ requires the simultaneous presence of high-frequency phonon modes, a large electron-phonon coupling, and a high electronic density of states (DOS) at the Fermi level (*N*_*F*_). These conditions are only partially met in most previously studied systems. For example, the *sp* ^2^-bonded carbon structures have high *N*_*F*_ values but only moderate to low electron-phonon coupling, while the *sp* ^3^-bonded diamond has high-frequency phonon modes and strong electron-phonon coupling but low *N*_*F*_ values^2, 18^. It has been predicted that diamond could reach higher *T*_*c*_ of up to 55 K with a 30% boron doping^19^, but such a high doping level is hard to achieve experimentally^20^. Theoretical efforts also explored materials with narrow electronic bands or reduced dimensionality to enhance *N*_*F*_ and obtained promising results, although material synthesis and stabilization remain challenging^21, 22^.

**Figure 1:**
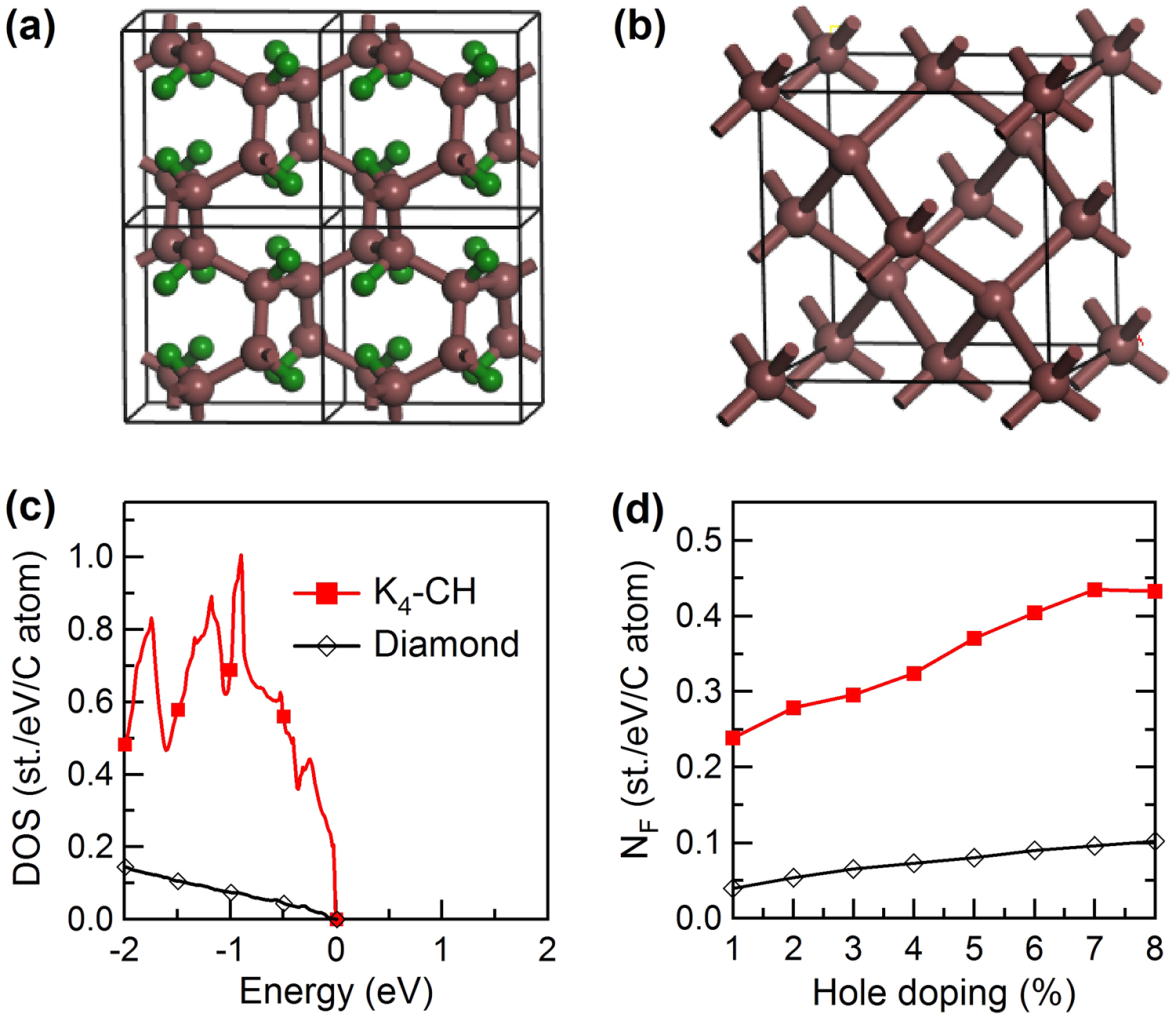
(**a**) Crystal structure of *K*_4_-CH (*I*2_1_/3), in comparison with that of (**b**) diamond (*Fd* $\bar{3}$ *m*). Large brown and small olive spheres represent the C and H atoms, respectively. The atomic Wyckoff positions are 8a (0.8384, 0.6616, 0.3384) C and 8a (0.1893, 0.3107, 0.6893) H for *K*_4_-CH. (**c**) Electronic density of states (DOS) and (**d**) *N*_*F*_ as a function of hole doping for *K*_4_-CH and diamond. The top of the valence bands is set as energy zero.

In this paper, we identify by *ab initio* calculations phonon-mediated high-*T*_*c*_ superconductivity in hole-doped cubic crystalline hydrocarbon *K*_4_-CH^23^ (space group *I*2_1_/3, named after the *K*_4_ carbon^24^). This structure comprises a strong *sp* ^3^-bonded carbon framework as in diamond [see Fig. 1(a)], which produces high-frequency phonon modes. Moreover, it also possesses a quickly rising electronic DOS near the top of the valence *σ*-band, allowing for a strong electron-phonon coupling. The presence of all three key properties required for phonon-mediated high-T_*c*_ superconductivity produces *T*_*c*_ values above 80 K in *K*_4_-CH at an experimentally accessible hole doping level of a few percent. These results promise to stimulate efforts for synthesis and further exploration of this novel superconducting covalent metal.

## Results

The structural detail of *K*_4_-CH is schematically depicted in Fig. 1(a). This hydrocarbon crystallizes in a body-centered cubic structure with equilibrium lattice parameter *a* = 4.250 Å. The calculated C-C bond length for *K*_4_-CH is 1.566 Å, close to 1.532 Å for diamond [Fig. 1(b)], indicating the saturated nature of the *sp* ^3^ carbon bonding in forming the three-dimensional (3D) covalent framework. Figure 1(c) shows the calculated electronic DOS for diamond and *K*_4_-CH. In diamond, the DOS increases slowly into the valence band. In stark contrast, in *K*_4_-CH, the DOS rises steeply from the valence band edge, reaching high values at low doping levels. Results in Fig. 1(d) show that at 3% hole doping the value of *N*_*F*_ for *K*_4_-CH reaches 0.30 states/eV/C, which are almost five times as high as the 0.065 states/eV/C for diamond. Such a significant DOS enhancement in the hydrocarbon is ascribed to the much smaller band dispersion in the proximity of the valence bands compared to diamond (see Fig. S2 in Supplementary Information).

**Figure 2:**
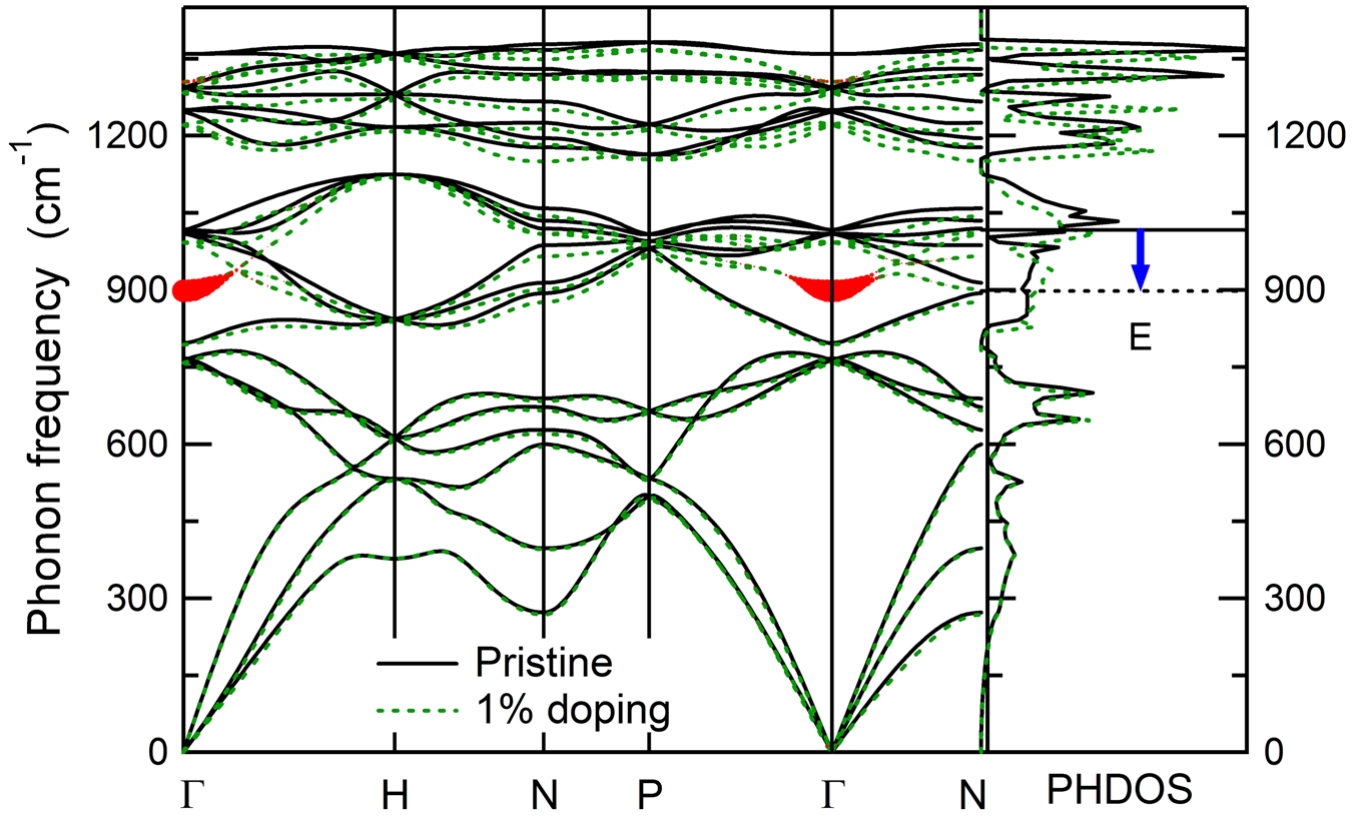
Phonon dispersion and PHDOS of pristine and 1% hole-doped *K*_4_-CH. The thickness of the red curves denotes the mode EPC strength *λ*_**q***ν*_ [Eq. (1)]. The softening of the optical zone-center modes is indicated by the blue arrow.

The phonon dispersion (*ω*_q*ν*_) and phonon density of states (PHDOS) calculated for pristine and hole-doped *K*_4_-CH are shown in Fig. 2. For pristine hydrocarbon, no imaginary phonon frequencies are found in the whole Brillouin zone, indicating its dynamical stability. In the presence of hole doping, the calculated optical phonon dispersions exhibit an obvious softening near the zone center, similar to the cases of doped diamond^18^ and graphane^22^.

**Figure 3:**
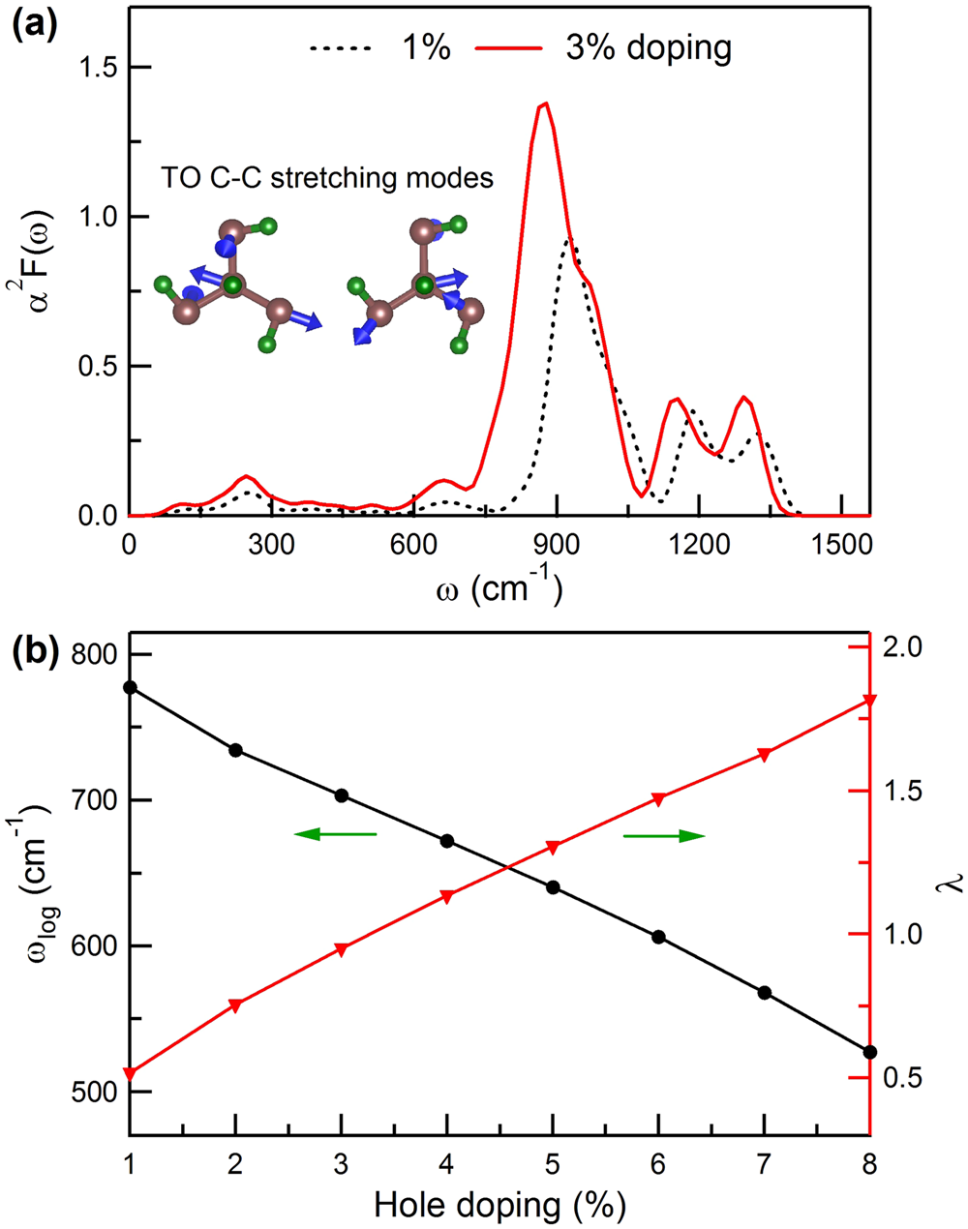
(**a**) Eliashberg spectral function *α* ^2^ *F*(*ω*) for *K*_4_-CH at 1% (black dotted line) and 3% (red solid line) doping. (**b**) Calculated *ω*_*log*_ (black dots) and *λ* (red triangles) as a function of hole doping for *K*_4_-CH. The insets in (**a**) show the characteristic TO C-C bond-stretching modes with the largest EPC contribution. The blue arrows indicate the atomic displacement patterns.

To gain insights into the influence of the optical phonon modes that dominate the EPC in hole-doped hydrocarbon, we evaluate the electron-phonon interaction *λ*_**q***ν*_ for a phonon mode *ν* with momentum **q** ^25^:

1 $${\lambda }_{{\bf{q}}\nu }=\frac{4}{{\omega }_{{\bf{q}}\nu }{N}_{F}{N}_{k}}\sum _{{\bf{k}},n,m}{|{g}_{{\bf{k}}n,{\bf{k}}+{\bf{q}}m}^{\nu }|}^{2}\delta ({\epsilon }_{{\bf{k}}n})\delta ({\epsilon }_{{\bf{k}}+{\bf{q}}m}),$$

where *ω*_**q***ν*_ is the phonon frequency, ${\epsilon }_{{\bf{k}}n}$ is the Kohn-Sham energy, and ${g}_{{\bf{k}}n,{\bf{k}}+{\bf{q}}m}^{\nu }$ represents the electron-phonon matrix element. It is seen from Fig. 2 that for *K*_4_-CH the two-fold degenerate optical *E* modes at Γ with the largest EPC are softened by 120 cm^−1^ at 1% doping. By inspecting the calculated vibrational pattern of the softened modes [see the insets of Fig. 3(a)], we find that these transverse optical (TO) zone-center phonon modes correspond to the C-C bond stretching modes, which couple considerably to the holes at the top of the *σ*-bonding valence bands. Furthermore, the softening of the TO C-C stretching modes (120 cm^−1^ at 1% doping) is comparable to the 138 cm^−1^ of diamond^18^ but significantly smaller than the 470 cm^−1^ of graphane^22^. This can be explained by the 3D nature of the carbon framework in *K*_4_-CH as in diamond, which has a weaker Kohn effect that reduces the mode softening compared to 2D graphane^22^. It is noted that the high-frequency C-H stretching modes are only weakly affected by the hole doping (see Fig. S3 in Supplementary Information), suggesting their small contributions to the EPC as in doped graphane^22^.

We next examine the doping induced superconductivity in the hydrocarbon. We show in Fig. 3(a) the calculated Eliashberg spectral function *α* ^2^ *F*(*ω*) for 1% and 3% hole-doped *K*_4_-CH, which describes the relative contribution to the EPC by different phonon modes^25^:

2 $${\alpha }^{2}F(\omega )=\frac{1}{2{N}_{q}}\sum _{{\bf{q}}\nu }{\lambda }_{{\bf{q}}\nu }{\omega }_{{\bf{q}}\nu }\delta (\omega -{\omega }_{{\bf{q}}\nu }\mathrm{)}.$$

As in doped diamond and graphane^18, 22^, the electron-phonon interaction here is also dominated by the coupling of the *σ* holes to the optical phonon modes, and only a small contribution comes from the acoustic phonon modes. There is a single dominant peak in the Eliashberg function for *K*_4_-CH, and the peak position corresponds to the frequency of the softened TO C-C stretching mode shown in Fig. 2. As the doping increases, the main peak of *α* ^2^ *F*(*ω*) moves toward lower phonon frequency, reflecting further softening of the characteristic optical mode; meanwhile, the intensity of the main peak is enhanced, resulting primarily from the sharp increase in *N*_*F*_ [see Fig. 1(d)], which allows more electronic states at the Fermi level to couple to the phonon modes and thus increases the EPC strength (*λ*_**q***ν*_) at the higher doping level as described by Eqs (1) and (2).

Using the Eliashberg functions, we have calculated the logarithmic average phonon frequency ${\omega }_{log}=\exp \,[\frac{2}{\lambda }\int d\omega {\alpha }^{2}F(\omega )\,\mathrm{ln}\,\omega /\omega ]$ and the EPC parameter *λ* = 2∫*dωα* ^2^ *F* (*ω*)/*ω*. The obtained results for hole-doped *K*_4_-CH are presented in Fig. 3(b). Clearly, with increased hole doping *ω*_*log*_ decreases as a result of the redshift of the optical mode that dominates the EPC, while *λ* increases mainly due to the increasing *α* ^2^ *F*(*ω*). The calculated *ω*_*log*_ at 3% doping is 703 cm^−1^ in *K*_4_-CH, smaller than the 1077 cm^−1^ in doped diamond^18^. The reduction in *ω*_*log*_ for the hydrocarbon is due to the lower frequency of the characteristic optical modes compared to diamond; they are located in the medium frequency region of the carbon framework optical modes (see Fig. 2), in contrast to diamond that possesses the highest optical zone-center phonon modes. The corresponding *λ* value of *K*_4_-CH is 0.95, considerably larger than the value of 0.30 at 3% calculated for hole-doped diamond^18^. Such large *λ* value compensates the relatively small *ω*_*log*_ to yield large *T*_*c*_ value in the doped hydrocarbon (see below).

**Figure 4:**
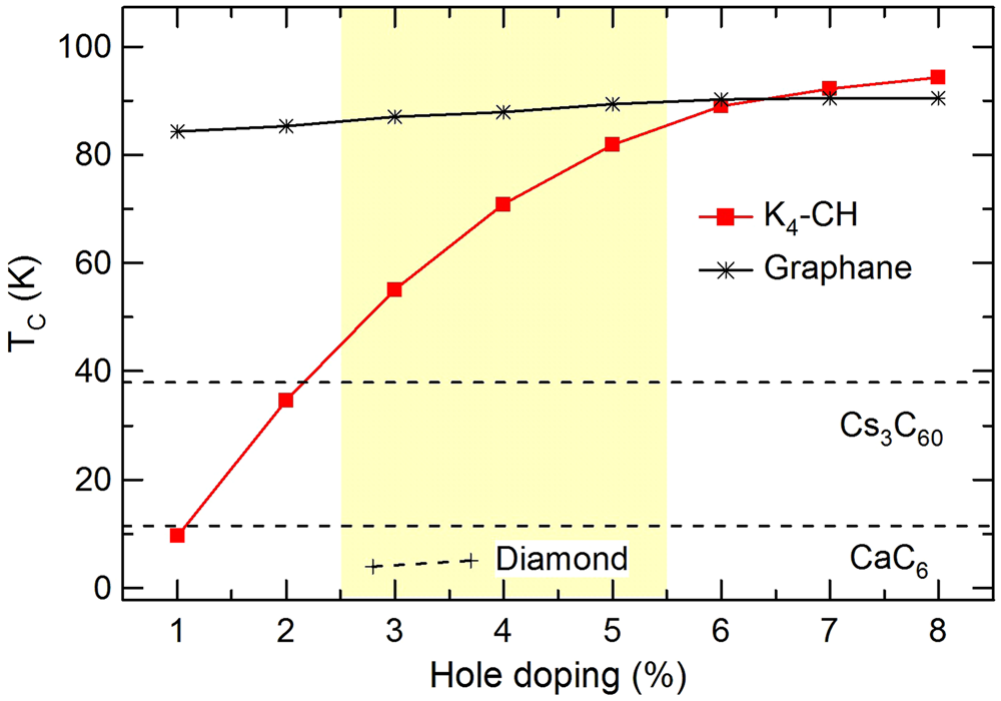
Calculated *T*_*c*_ as a function of hole doping for *K*_4_-CH. The experimental *T*_*c*_ (dashed black lines) of Cs_3_C_60_ (38 K^9^), CaC_6_ (11.5 K^6^), and diamond (4 K at ∼2.8% boron^14^; 5 K at ∼3.7% boron^15^), and the theoretical *T*_*c*_ (solid black line) of hole-doped graphane^22^ are shown for comparison. The shaded region indicates the experimentally accessible doping levels^16^.

Figure 4 shows the superconducting transition temperature *T*_*c*_ calculated for hole-doped *K*_4_-CH using the modified McMillan equation^26^:

3 $${T}_{c}=\frac{{\omega }_{log}}{1.2}\exp \,[\frac{-\mathrm{1.04(1}+\lambda )}{\lambda -{\mu }^{\ast }\mathrm{(1}+0.62\lambda )}],$$

with a screened Coulomb pseudopotential μ* = 0.13 (a typical value falling in the range 0.10–0.15 for most carbon-based materials^27–29^). It is seen that *T*_*c*_ increases with rising hole doping. At 3% doping, the calculated *T*_*c*_ is 55.1 K in *K*_4_-CH, which is significantly larger than the *T*_*c*_ of 4–5 K in boron-doped diamond at comparable doping levels^14, 15^. More remarkably, *K*_4_-CH exhibits very high *T*_*c*_ values above 80 K at 5% doping and becomes competitive with the previously studied hole-doped graphane^22^. It is important that such large *T*_*c*_ values are achieved at the experimentally accessible low doping levels, making it feasible to realize phonon-mediated high-*T*_*c*_ superconductivity in this hydrocarbon.

We now comment on the synthesis and doping of the hydrocarbon studied here. Superconducting boron-doped diamond was first produced under high-pressure and high-temperature conditions^14^. Experimental evidence has shown pressure-induced polymerization of acetylene or benzene into saturated CH structures^30, 31^, and this method is promising for synthesizing the *sp* ^3^-bonded hydrocarbon superconductor through high-pressure treatments of the molecular precursors with the incorporation of boron hydrides. An alternative approach is the chemical vapor deposition growth of boron-doped CH films, which has been widely used in preparing diamond-like CH films from different precursors^32, 33^. Compared with the high-pressure synthesis, growing the superconducting doped hydrocarbon films in such a nonequilibrium way can yield an increase in the boron concentration and thus a larger *T*_*c*_, as was demonstrated in the case of boron-doped diamond^14–16^.

## Discussion

In summary, we have shown by *ab initio* calculations that hole-doped *sp* ^3^-bonded covalent hydrocarbon *K*_4_-CH is a promising candidate for showing high-*T*_*c*_ superconductivity mediated by the electron-phonon coupling. The key advantages of this system lie in its quickly rising electronic DOS upon doping, together with the presence of high-frequency optical bond-stretching phonon modes and their strong coupling to the holes in the *σ*-band valence states. At the experimentally accessible doping range of up to 5%^16^, *T*_*c*_ in crystalline hydrocarbon *K*_4_-CH can reach 80 K. Our results identify a new carbon-based covalent metal as extraordinary electron-phonon superconductor, and the insights offered by the calculated structural, phonon, and electronic properties lay the foundation for further exploration of this material.

## Methods

The calculations are performed using the density functional theory within the local density approximation (LDA)^34, 35^, as implemented in the Quantum ESPRESSO code^36^. Norm-conserving pseudopotentials^37^ are adopted with a plane-wave cutoff energy of 100 Ry. We employ the virtual crystal approximation^18^ to simulate the hole doping by generating B_*x*_C_1−*x*_ pseudopotentials with *x* = 0.01–0.08. Its feasibility was proved by comparing the electronic structure with the supercell model explicitly including the boron atoms (see Fig. S1 in Supplementary Information). The dynamical matrices and the electron-phonon coupling (EPC) are calculated using the density functional perturbation theory^38^. The phonon dispersion is obtained by the Fourier interpolation of the dynamical matrices computed on a *N*_*q*_ = 6 × 6 × 6 **q**-mesh. For the electronic integration in the phonon calculation, we use a *N*_*k*_ = 12 × 12 × 12 **k**-mesh and a Methfessel-Paxton^39^ smearing of 0.02 Ry. A finer *N*_*k*_ = 24 × 24 × 24 **k**-mesh is used for obtaining the EPC and the electronic DOS. Such samplings ensure good convergence of phonon frequencies and the average coupling *λ*. We have also used the generalized gradient approximation (GGA)^40^ for electron-phonon calculations to confirm our results for superconductivity in the hole-doped hydrocarbon (see Table S1 in Supplementary Information).

## Electronic supplementary material

Supplementary Material
