# Supplementary material for: Phonon-mediated high-Tc superconductivity in hole-doped diamond-like crystalline hydrocarbon
Source: Sci Rep. 2017 May 3;7:1464. doi: 10.1038/s41598-017-01541-6 (PMC5431101; doi:10.1038/s41598-017-01541-6)
Supplement: Supplementary file 1 — Supplementary Material [file 41598_2017_1541_MOESM1_ESM.doc]

**Supplementary information**

**Phonon-mediated high-T_c_ superconductivity in hole-doped diamond-like crystalline hydrocarbon**

Chao-Sheng Lian^1^, Jian-Tao Wang^1,2^,*, Wenhui Duan^3^, and Changfeng Chen^4^

^1^Beijing National Laboratory for Condensed Matter Physics,Institute of Physics, Chinese Academy of Sciences, Beijing 100190, China

^2^School of Physics, University of Chinese Academy of Sciences, Beijing 100049, China

^3^Department of Physics and State Key Laboratory of Low-Dimensional Quantum Physics, Tsinghua University, Beijing 100084, China

^4^Department of Physics and High Pressure Science and Engineering Center, University of Nevada, Las Vegas, Nevada 89154, USA

1. **Virtual crystal approximation (VCA) for the hole-doped hydrocarbon [Fig. S1]**

Figure S1(a) shows a comparison between the calculated electronic DOS of hole-doped K_4_-CH within the VCA model and the supercell model with substitutional boron doping. We used a 2×2×2 supercell with a carbon atom substituted by a boron atom, corresponding to 3.2% hole doping (a 1% doping refers to one B every 100 C). We see in both cases that the doping has the effect of lowering the Fermi energy E_F_ below the top of the valence bands, and in particular for the supercell model that the dopants do not introduce localized states in the band gap, as evidenced also by the calculated atomic-orbital projected DOS with a Gaussian smearing of 0.007 Ry shown in Fig. S1(b). Importantly, the DOS at E_F_ is found to be similar in these two models, providing a justification for the virtual crystal approximation to simulate the substitutional doping in the present work.


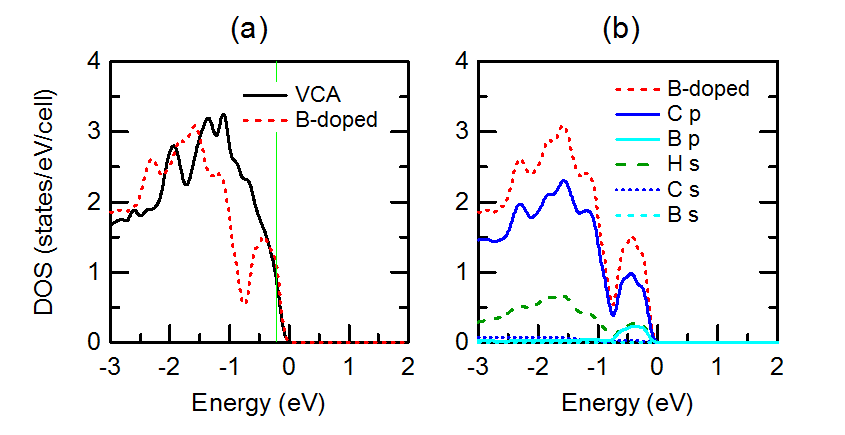


FIG. S1: (a) Electronic DOS of the 3.2% hole-doped K_4_-CH calculated within the VCA model (solid black line) and the supercell model (dashed red line, 2×2×2 supercell with boron). The top of the valence bands is set as energy zero, and E_F_ is -0.23 eV (vertical green line). (b) Atomic-orbital projected DOS of the boron-doped K_4_-CH.

1. **Electronic properties of the pristine hydrocarbon [Fig. S2]**

The calculated electronic band structure and atomic-orbital projected DOS (with 0.007 Ry Gaussian smearing) of K_4_-CH are shown in Fig. S2(a). In comparison with diamond [Fig. S2(b)], the band dispersion in the proximity of the valence bands is much smaller for K_4_-CH, which stems from the electronic state hybridization between the C and H atoms [Fig. S2(a)] that frustrates the formation of bonding *p*-states at the valence band top and eventually leads to the reduced band dispersion. It is also noted for K_4_-CH that the electronic states near the top of the valence bands are dominated by the C-*p* characters, suggesting that upon doping the holes will be mainly introduced in the electronic states associated with the C-C bonds which can couple strongly to the corresponding bond-stretching phonons.


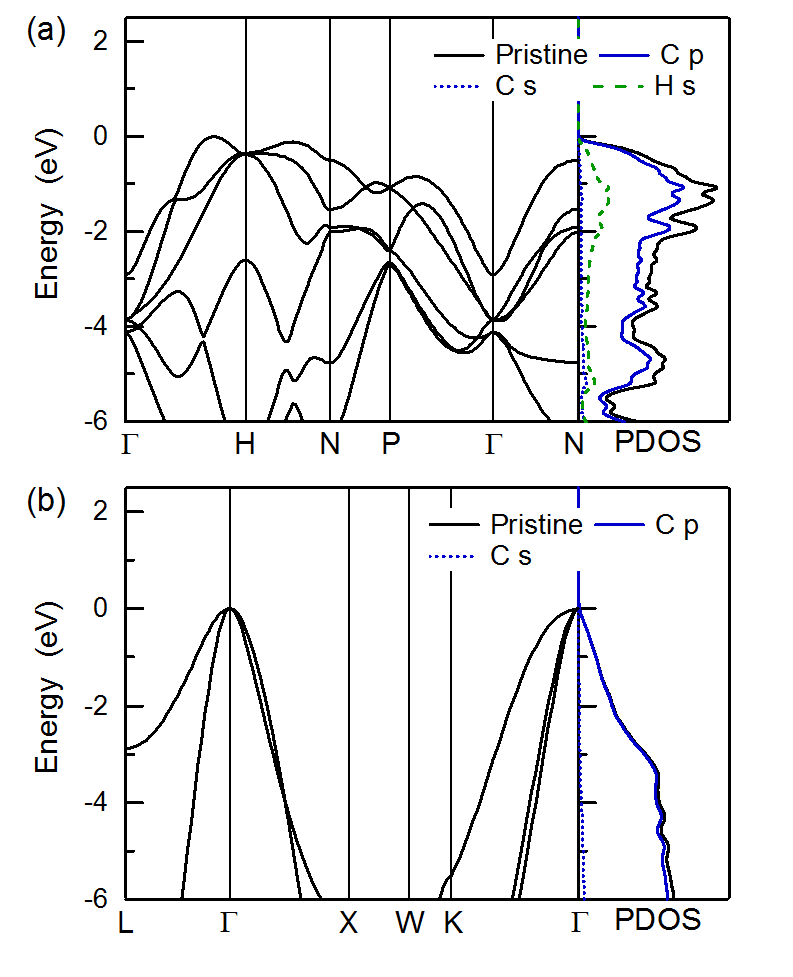


FIG. S2: Electronic band structure and atomic-orbital projected DOS of (a) K_4_-CH and (b) diamond. The top of the valence bands is set as energy zero.

1. **High-frequency phonon dispersions [Fig. S3]**

The calculated high-frequency phonon dispersions for the pristine and 1% hole-doped K_4_-CH are shown in Fig. S3. This hydrocarbon has four C-H stretching modes, consistent with the number of C-H bonds in the primitive cell. It is seen that the dispersions of the high-frequency C-H stretching modes are only weakly affected by the hole doping. At the Γ point, the largest mode softening at 1% doping is 15 cm^-1^ in K_4_-CH. By integrating the calculated Eliashberg spectral function at 1% doping in the high-frequency range, we find the contribution to the total electron-phonon coupling (EPC) strength to be 4.3% for K_4_-CH. These results indicate that the C-H stretching modes have small weights on the EPC, consistent with the fact that only a small contribution to the Fermi level DOS comes from the electronic states associated with the C-H bonds.


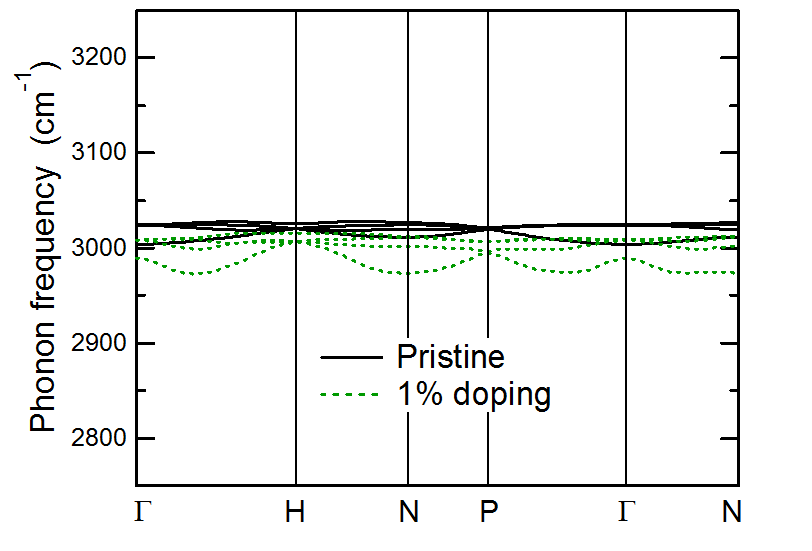


FIG. S3: Dispersions of the high-frequency C-H stretching modes for the pristine and 1% hole-doped K_4_-CH.

1. **Comparison between LDA and GGA results**

Table S1 shows the calculated structural and electron-phonon properties under both LDA and GGA for K_4_-CH. It is seen that the Fermi level DOS N_F_ at 3% doping increases from LDA to GGA. Consistent with the variation in N_F_, the corresponding λ value is found to be enhanced in K_4_-CH at the GGA level. As a consequence, from LDA to GGA T_c_ at 3% doping increases from 55.1 to 66.0 K. These results confirm the validity of our conclusions for superconductivity in the hole-doped hydrocarbon.

Table S1: Lattice parameter *a* (Å) and the Fermi level DOS N_F_ (states/eV/C) at 3% hole doping calculated within LDA and GGA for K_4_-CH. The EPC parameter λ, logarithmic average phonon frequency ω_log_ (cm^-1^), and T_c_ (K) obtained at 3% hole doping are also given.

| Method | Lattice parameter | 3% hole doping | | | |
| --- | --- | --- | --- | --- | --- |
|  | *a* | N_F_ | λ | ω_log_ | T_c_ |
| LDA | 4.250 | 0.296 | 0.950 | 703.1 | 55.1 |
| GGA | 4.319 | 0.315 | 1.050 | 706.7 | 66.0 |
